# Lateralized Neural Responses to Letters and Digits in First Graders

**DOI:** 10.1111/cdev.13337

**Published:** 2019-10-27

**Authors:** Aliette Lochy, Christine Schiltz

**Affiliations:** ^1^ University of Luxembourg

## Abstract

The emergence of visual cortex specialization for culturally acquired characters like letters and digits, both arbitrary shapes related to specific cognitive domains, is yet unclear. Here, 20 young children (6.12 years old) were tested with a frequency‐tagging paradigm coupled with electroencephalogram recordings to assess discrimination responses of letters from digits and vice‐versa. One category of stimuli (e.g., letters) was periodically inserted (1/5) in streams of the other category (e.g., digits) presented at a fast rate (6 Hz). Results show clear right‐lateralized discrimination responses at 6 Hz/5 for digits within letters, and a trend for left‐lateralization for letters. These results support an early developmental emergence of ventral occipito‐temporal cortex specialization for visual recognition of digits and letters, potentially in relation with relevant coactivated brain networks.

Learning to read written symbols induces changes in the visual cortex, leading to selective neural population responses to letters or digits in comparison to other visual patterns (Park, Chiang, Brannon, & Woldorff, 2014). This cultural impact on the organization of the visual system impressively reveals the role of experience in shaping the brain’s functional organization. Letters and digits are arbitrary symbols (i.e., bearing no relation between shape and meaning) with visually similar 2D patterns acquired through explicit instruction. However, they belong to different cognitive and semantic domains: language and numbers. An outstanding challenge is to understand how and when the initially meaningless shapes turn into sophisticated and highly differentiated semantic representations. Here we investigated whether letters (alphabetic characters) and numbers (Arabic digits) already give rise to distinct neural responses in young children who just started primary school.

Letters relate to phonology and are the building blocks of written words, which represent oral language, whereas Arabic digits relate to numerical knowledge: they represent numerosity and correspond to verbal number words. For letters and words, the left ventral occipito‐temporal cortex (VOTC), and more specifically the mid‐fusiform gyrus, is preferentially recruited in literate adults (McCandliss, Cohen, & Dehaene, 2003; Schlaggar & McCandliss, 2007). This “visual word form area” (VWFA) has been proposed to specialize ontogenetically for representing strings of letters (McCandliss et al., 2003), even though its specificity has been questioned (Price & Devlin, 2003, 2011). Specialization for letter stimuli is congruent with the general principles of the VOTC organization, showing high category‐specificity for visual stimuli such as faces or body parts (Grill‐Spector & Weiner, 2014).

For Arabic digits, results are more controversial: there is no consensus yet on the existence of a specific neural network for their visual processing in numerate adults (for review, Yeo, Wilkey, & Price, 2017). The Triple‐code model (Dehaene, 1992; Dehaene & Cohen, 1995) postulates a specific representation for Arabic digits: the *Visual Arabic Number Form* code represents numbers as an ordered string of digits used to support specific numerical operations (e.g., calculation), and is processed in bilateral VOTC within general object recognition regions. This proposal implies the existence of a region in the VOTC that would be more engaged by Arabic numerals than any other written characters (“Number Form Area”—NFA). Given the functional dissociations between letters and numbers recognition in cases of alexia (Starrfelt & Behrmann, 2011), neuropsychological reports suggested that they might be processed by distinct neural structures. However, such dissociations always revealed a weaker impairment for digits than letters, leading to the alternative view that digits would simply be easier to recognize and therefore, more resistant to brain damage (Schubert, 2017; Starrfelt & Behrmann, 2011).

So far, functional MRI (fMRI) studies of visual processing of Arabic numbers have not provided clear answers to this issue, finding no number‐specific region (Price & Ansari, 2011; Reinke, Fernandes, Schwindt, O’Craven, & Grady, 2008), or activation bilaterally (Grotheer, Herrmann, & Kovacs, 2016), in left (Fias, Lammertyn, Caessens, & Orban, 2007) or right VOTC (Pinel, Dehaene, Rivière, & LeBihan, 2001). Finally, a substantial amount of studies found activations only outside of the VOTC (Yeo et al., 2017 for a review). Conclusions on fMRI studies are limited by the fact that the NFA might be located within a region where BOLD signal is difficult to record due to magnetic susceptibility artefacts (Shum et al., 2013) and possibly varies with (mathematical) task demands (Grotheer, Jeska, & Grill‐Spector, 2018; Peters, De Smedt, & Op de Beeck, 2015).

Directly contrasting passive viewing of Arabic digits and meaningless letter‐strings, an fMRI study observed greater activation for letters in the left VOTC (mid‐fusiform gyrus and inferior temporal gyrus) and for digits in the right VOTC (lateral occipital cortex; Park, Hebrank, Polk, & Park, 2011). Interestingly, the latter related to lateralization of responses in the intraparietal sulcus (IPS) for numerical tasks on nonsymbolic stimuli (i.e., addition, subtraction, counting). The authors interpreted this result as a possible mechanism by which higher level numerical tasks might constraint the categorical organization in the VOTC. Finally, the temporal characteristics of this letter/digits dissociation was investigated using event related potentials (ERPs) (Park, van den Berg, Chiang, Woldorff, & Brannon, 2017; Park et al., 2014). Different amplitude response patterns were found for letters and numbers in early electrophysiological components, the N170 being left‐lateralized for letters, and right‐lateralized for Arabic digits. Thus, in adults, early visual processing of digits and letters seems to be segregated as a function of codes.

In children, studies on the neuronal substrate of (differential) letter and digit processing are scarce (Cantlon, Pinel, Dehaene, & Pelphrey, 2011; Libertus, Brannon, & Pelphrey, 2009). Recently, Park et al. (2017) examined ERP responses (N1‐latency amplitudes) to letters or digits in a cross‐sectional developmental study. Surprisingly, results revealed a late developmental differentiation in the neural responses to letters and digits: at 7 years old, no lateralization and no differentiation emerged between codes. At 10 years old, letters tended to elicit greater responses than digits, but in both hemispheres. Only at 15 years old was the response pattern lateralized similarly to adults. This result is particularly surprising because children are familiarized with letters and numbers at least from age three onwards (Wright, England, & Rivers, 1991). The authors concluded that visual exposure and rudimentary knowledge is not sufficient for tuning the visual cortex finely to these two categories of characters.

We would like to revisit and challenge this conclusion using a sensitive “frequency‐tagging” or fast periodic visual stimulation electroencephalogram (EEG) approach to reveal specific visual discrimination signals (e.g., Liu‐Shuang, Norcia, & Rossion, 2014; Rossion, Torfs, Jacques, & Liu‐Shuang, 2015). In this paradigm, various base stimuli are presented at a fast rate (e.g., pseudofonts at 6 Hz), and stimuli from a specific category (e.g., words) are periodically inserted in the stream (e.g., 1 every 5, thus at 1.2 Hz). If words systematically elicit a specific (i.e., differential) response, it occurs exactly at this stimulation frequency (i.e., 1.2 Hz). Importantly, there is no need of performing a post hoc contrast between two conditions (e.g., response to words *minus* response to pseudofonts), since the response itself is an index of differential processing. This approach was successfully used to demonstrate a left hemisphere (LH) specialization for letters in preschool children, (Lochy, Van Reybroeck, & Rossion, 2016) whereas standard ERP studies had suggested this change to occur after 1–1.5 years of schooling (Eberhard‐Moscicka, Jost, Raith, & Maurer, 2015; Maurer et al., 2006).

We used this sensitive approach to examine in first graders whether neural responses specific to single letters (LETT) and digits (DIG) could already be elicited, when inserted in streams of the other category. This direct contrast allowed to obtain specific responses for letters within streams of digits (hereafter: dig‐LETT) and vice‐versa (hereafter: lett‐DIG).

If letters and digits already elicit specialized neural processes at 6 years of age, we should observe responses at the frequency of the categorical change. For dig‐LETT, we expected a left‐lateralized posterior response, as letters are visual objects learnt directly in association with phonological codes and trigger connections between anterior phonological and posterior visual regions (Phonological Mapping Hypothesis, Maurer & McCandliss, 2007). For lett‐DIG we anticipated a right response lateralization, given the previous findings on adults (Park et al., 2017, 2014) and the potential link with lateralization for numerical competencies heavily relying on the right IPS (rIPS; Cantlon, Brannon, Carter, & Pelphrey, 2006; Vogel, Goffin, & Ansari, 2015),

## Method

### Participants

Twenty first‐grade children (10 males, *M*_age_ = 6.12 years; range = 5.11–7.03 years) with normal or corrected‐to‐normal vision, were tested after parents gave their written informed consent for a study approved by the Ethical Committee of the Catholic University of Louvain. Participants were recruited from two different schools in Brussels, and had been enrolled in French‐speaking school since kindergarten. Three participants were excluded because of abnormal scores in more than two behavioral assessments in the screening battery for general cognitive functions and reading (Table 1). Ethnicity of children was as follows: 8 Caucasian, 10 Arabic, 2 African (Table 1 for demographic information, typical of this area).

**Table 1:** Descriptive Statistics for Demographic Characteristics and Tests of General Cognitive Functions and Reading (*N* = 17)

	Scores		
	Min	Max	*M* (*SD*)
Behavioral tests			
Perceptual reasoning (CPM, chance‐level 16%, accuracy in %)	55.56	83.33	70.59 (8.39)
Selective attention (TEA‐Ch, speed in sec)	5.20	21.94	9.67 (4.37)
Vocabulary production (N‐EEL, accuracy in %)	31.58	84.21	67.39 (12)
Reading (BELO, accuracy in %)	5.64	39.18	20.18 (11.04)
Demographic characteristics			
Parents’ maximum education level (1: primary school, 2: Middle school, 3: High school, 4: Bachelor, 5: Graduate degree)	1	5	2.73 (1.50)
5 French monolinguals, 12 bilinguals: L2 Arabic (6), Polish (2), Spanish (1), Romanian (1), Bangla (1), and 3 trilinguals (L2 Arabic, L3 English)			

### EEG Testing Stimuli

**Figure 1:**
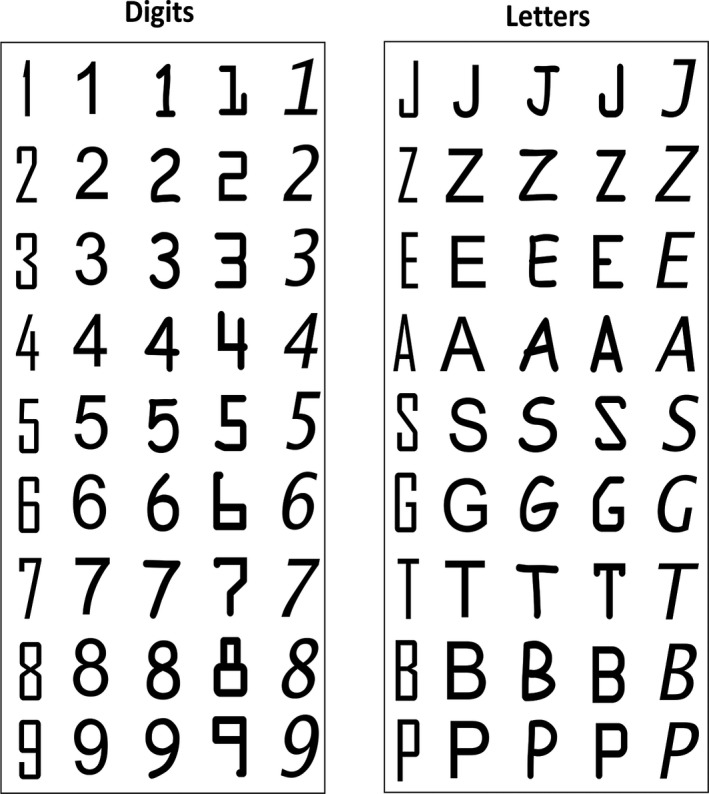
Stimuli. Digits and matched upper‐case letters in five different fonts (left to right: Agency FB, Arial, Comic sans MS, OCR A extended, Lucida sans).

The nine possible Arabic digits (excluding 0) were presented in five different fonts (Figure 1). Nine upper‐case letters were chosen to match digits visually (overall shape and/or number of strokes) and were also presented in five different fonts (Figure 1). These fonts were used to maximize variability among the stimuli of a given category, in order to ensure that any selective response reflects generalization beyond specific visual features. Final images were resized to 236 × 236 pixels. At a distance of 1 m, displayed with an 800 × 600 pixel resolution, the average size was 6 × 6 degrees of visual angle. Images were presented at the center of the screen with no immediate repetition of the same stimulus.

### Procedure

**Figure 2:**
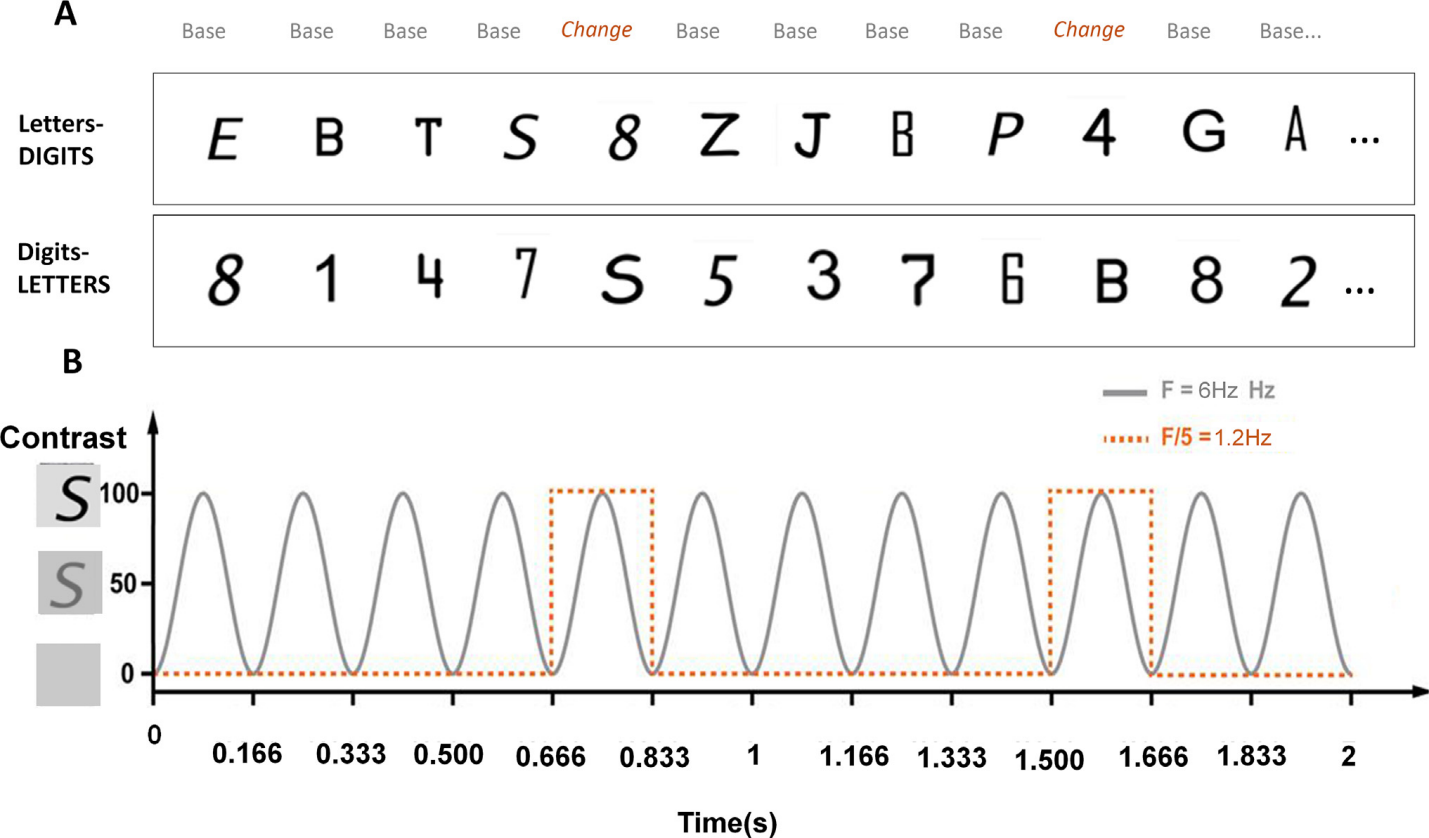
Experimental design. (A) Example of stimulation sequences. In the letters‐DIGITS condition, base stimuli consist of nine letters (in 5 fonts), randomly mixed, and digits are inserted every five items. The reverse occurs in the digits‐LETTERS condition. (B) Stimulation mode: six stimuli/second were presented with a sinusoidal contrast modulation, the categorical change occurred at 6 Hz/5 = 1.2 Hz. Two trials of 40 s were recorded per sequence type. [Color figure can be viewed at http://wileyonlinelibrary.com]

As in previous studies (Lochy et al., 2016), each stimulation sequence started with a fixation dot (2–5 s), 2 s of gradual stimulation fade in, 40 s of stimulation sequence, and 2 s gradual fade out (Figure 2). Stimuli were presented by means of sinusoidal contrast modulation at a base frequency rate of 6 Hz (i.e., every 166.66 ms; Figure 2B) with a software running over a JavaScript (Java SE Version 8, Oracle Corporation, Redwood Shores, CA, USA), and fonts randomly changed at every stimulation cycle.

Every sequence had the same structure: stimuli of the base category were presented at 6 Hz, and every fifth item was a stimulus of the other category (at 1.2 Hz, thus every 833 ms) such as DDDDLDDDDL … and reverse (Figure 2A). Within each category, each of the nine stimuli was presented an equal number of times. The sequence was repeated once for a total of 2 × 40 s. A pause was done between each of the sequences, which were initiated manually to ensure low‐artifact EEG signals.

To maintain a constant level of attention throughout the stimulation, children were instructed to fixate a central dot and detect brief color‐changes (200 ms, blue to red, 6 random changes per sequence) by pressing the space bar. Children performed almost at ceiling (91%–95%), and without differences between conditions in response times, dig‐LETT: 650 ms; let‐DIG: 665 ms [*F*(1, 16) < 1].

### EEG Acquisition and Preprocessing

Children were seated comfortably at 1 m from the computer screen in a quiet room of the school. EEG was acquired at 1024 Hz using a 37‐channel Biosemi Active II system (Biosemi, Amsterdam, The Netherlands), with 32 channels at standard 10‐20 system locations plus a row of posterior electrodes including PO9, I1, Iz, I2, PO10. The magnitude of the offset of all electrodes, referenced to the common mode sense, was held below 50 mV. EEG analyses were carried out using Letswave 5 (http://nocions.webnode.com/letswave), and Matlab 2012 (The MathWorks, Inc., Natick, MA, USA). After Fast Fourier Transform (FFT) band‐pass filtering between 0.1 and 100 Hz, data files were resampled to 512 Hz and segmented 2 s before and after each sequence, resulting in 44‐s segments. This allowed better visualization of the epochs for artifact/noise detection and correction with linear interpolation (3.9% of channels), before rereferencing to the common average. EEG recordings were then segmented again from stimulation onset until 39.996 s, corresponding to the largest amount of complete cycles of 833 ms (48 cycles) at the 1.2 Hz frequency within the 40 s of stimulation period.

### Frequency Domain Analysis

Per condition, the two trials were averaged in the time domain for each participant, in order to increase signal‐to‐noise ratio (SNR). A FFT was applied to the averaged time‐window, and normalized amplitude spectra were extracted for all channels. This yielded EEG spectra with a high‐frequency resolution (1/39.996 s = 0.025 Hz), increasing SNR and allowing unambiguous identification of the response at the exact frequencies of interest (i.e., 6 Hz for the base stimulation rate and 1.2 Hz and harmonics for the categorical change). To estimate SNR across the EEG spectrum, amplitude at each frequency bin was divided by the average amplitude of 20 surrounding bins (10 on each side; Liu‐Shuang et al., 2014). To quantify the responses of interest in microvolts, the average voltage amplitude of the 20 surrounding bins (i.e., the noise) was subtracted out (Retter & Rossion, 2016).

Based on the grand‐averaged amplitude spectrum for each condition, *Z*‐scores were computed at every channel to assess the significance responses at each stimulation frequency (base‐6 Hz, categorical change‐1.2 Hz) and harmonics (Liu‐Shuang et al., 2014; Lochy, Van Belle, & Rossion, 2015). *Z*‐scores were calculated for each discrete frequency bin (*x*) according to the formula *Z *= (*x*‐noise mean)/(noise standard deviation), where the noise was defined as the twenty frequency bins surrounding each target bin excluding the immediately adjacent bins and the local maximum and minimum amplitude bins. *Z*‐Scores larger than 1.64 (*p* < .05, one‐tailed, signal > noise) were considered significant.

To quantify the periodic response distributed on several harmonics, the baseline‐subtracted amplitudes of significant harmonics (excluding the base stimulation frequency) were summed for each participant, task, and condition, following the approach validated for the quantification of frequency‐locked responses in scalp studies (Retter & Rossion, 2016).

## Results

### Base Rate Responses

Responses at the base rate (6 Hz) were significant up to seven harmonics (42 Hz). As in previous studies (Lochy et al., 2016), we first summed the baseline‐corrected amplitudes and ranked the responses of the 37 electrodes. The six channels with the highest responses were all located in the occipital medial region (from 2.7 µV (Iz, I1) to 2.09 µV (Oz)), and were averaged in a region‐of‐interest (ROI). The effect of *Conditions* (lett‐DIG vs. dig‐LETT) was analyzed with a repeated measures analysis of variance (ANOVA). There was no significant effect of *Conditions, F*(1, 16) = 2.021; *MSE* = 0.187; *p* = .18.

### Categorical Discrimination Responses

**Figure 3:**
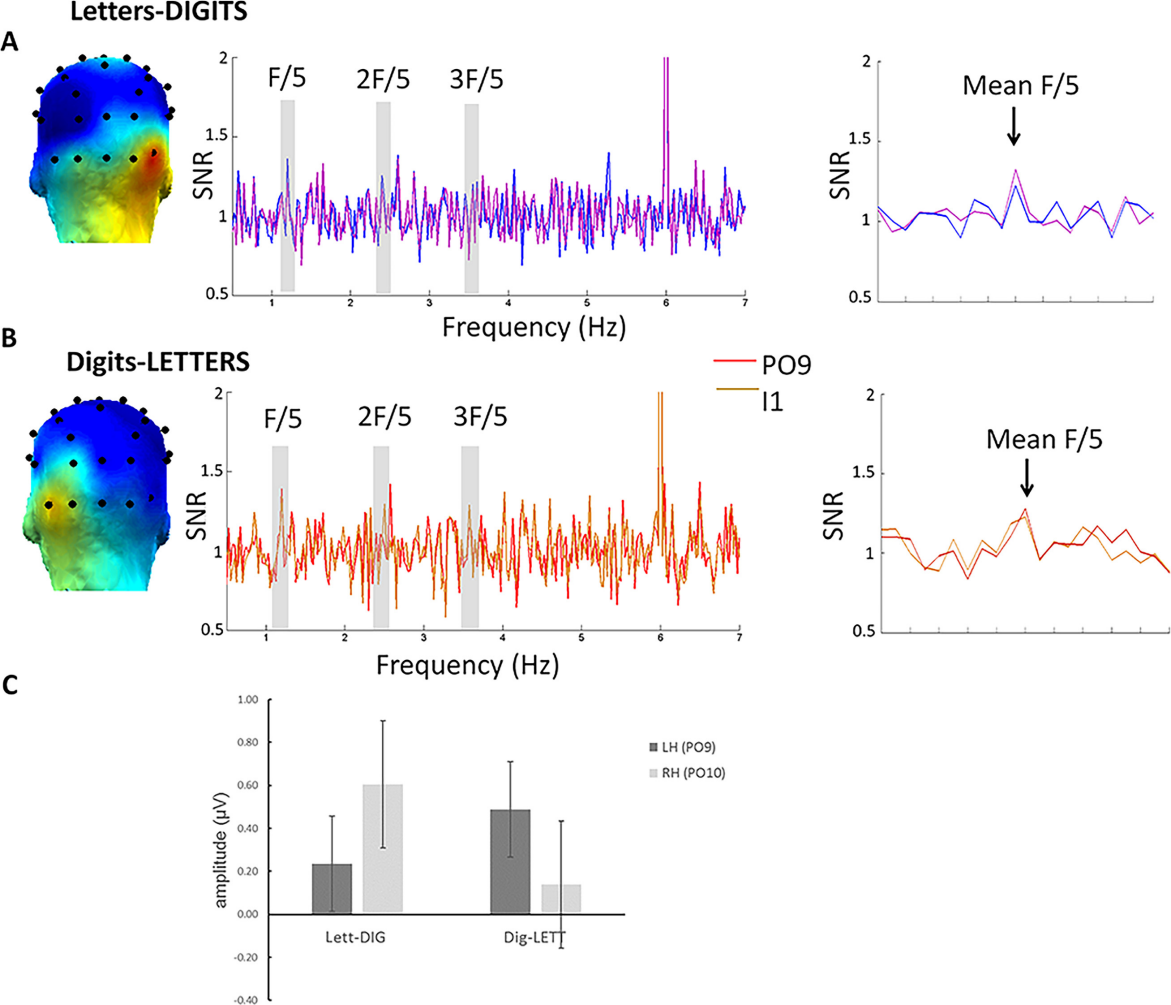
Discrimination responses for digits among letters and vice‐versa. (A and B) Topographies and SNR response spectra for letters‐DIGITS (A) and digits‐LETTERS (B). Each spectrum displays the two best electrodes for each condition, PO10, I2 for digits and PO9, I1 for letters. On the response spectra, the frequencies of significant (*Z* > 1.64) categorical responses over both conditions are marked as F/5 (1.2 Hz), 2F/5 (2.4 Hz) and 3F/5 (3.8 Hz). The response at 6 Hz represents base response at the sequence stimulation frequency. On the right side of the spectra, the averaged SNR of three significant harmonics is represented centered with 10 surrounding bins on each side. Topographies display the amplitude of discrimination responses color‐scaled between 0 µV (blue) to 0.61 µV (red) C. Mean amplitude values (in µV) and *SD* of discrimination responses, in each hemisphere (LH: dark gray, RH: light gray) per condition, giving rise to a significant interaction between *Hemispheres* and *Conditions*. RH = right hemisphere; LH = left hemisphere; LETT = letters; DIG = digits. [Color figure can be viewed at http://wileyonlinelibrary.com]

Discrimination responses were significant from 1.2 Hz up to 3.6 Hz (three harmonics). Scalp topographies of the sum of harmonics suggested a LH response for letters in digits stream, and a right hemisphere (RH) response for digits in letters stream (Figure 3). To allow comparison with previously published results, we selected electrodes of interest based on the study of Park (Park et al., 2017) that used 64 channels, where PO9‐PO7 were analyzed for the LH and PO10‐PO8 for the RH. Since we used a 32 channels system + 5 posterior electrodes, we reasoned that P7 was topographically too far from PO9 to include it in a ROI. Therefore, we performed our analyses on PO9 (LH) and PO10 (RH). Means and 95% confidence intervals (CI) are reported in the text, standard deviations are found in Figure 3B.

**Figure 4:**
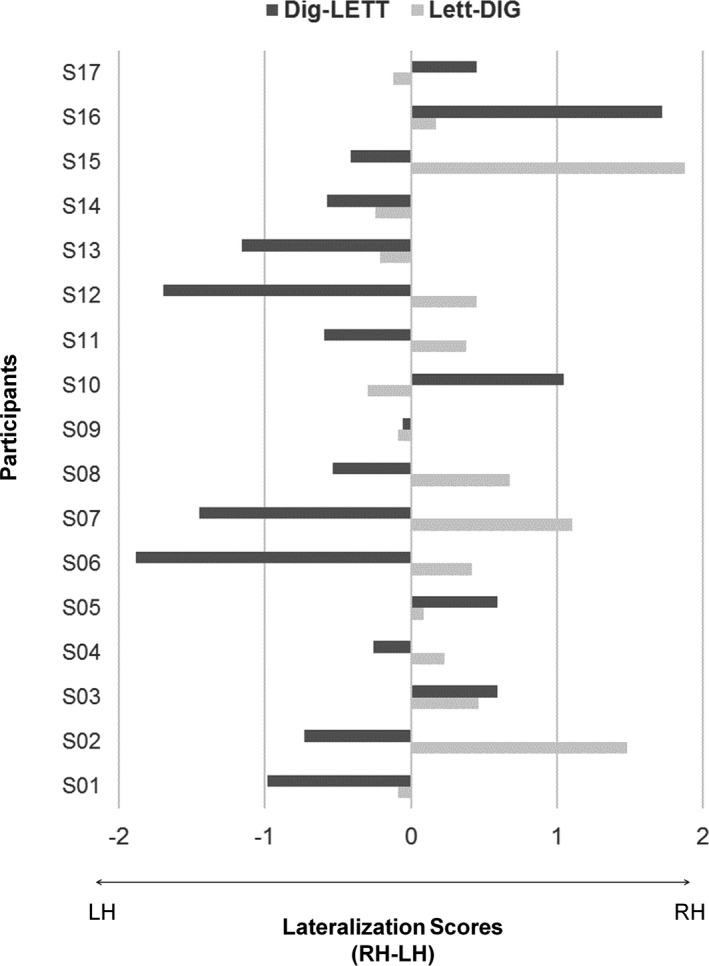
Individual data for lateralization scores in each condition. Amplitudes responses in the left hemisphere (PO9) were subtracted from responses in the right hemisphere (PO10). Positive values represent right‐lateralized responses, and negative values, left‐lateralized responses. RH = right hemisphere; LH = left hemisphere; LETT = letters; DIG = digits.

The sum of baseline‐corrected amplitudes for the three significant harmonics were submitted to a 2 (*condition*s: lett‐DIG vs. dig‐LETT) × 2 (*hemisphere*s: PO9 vs. PO10) repeated measures ANOVA. Main effects were not significant (*F* < 1), but there was a significant Conditions × Hemispheres interaction, *F*(1, 16) = 5.204; *MSE* = 2.196; *p* = .037 (Figure 3C). Paired sample *t*‐tests contrasting hemispheres revealed that for digits, response in the RH (*M* = .61 µV, CI [0.16, 1.053]) was significantly stronger and more reliable than in the LH (*M* = .23 µV; CI [−0.08, 0.56], *t*(16) = −2.466; *p* = .025; Figure 3A). For letters, we observed a trend for stronger responses in the LH (*M* = .49 µV; CI [0.019, 0.957]) than in the RH (*M* = .14 µV; CI [−0.49, 0.77], *t*(16) = 1.465; *p* = .16; Figure 3B). Variability in individual lateralization scores was somewhat greater for letters (5/17 participants with right lateralization, see Figure 4 for individual data).

## Discussion

This study found that 6‐year‐old children display specific neural responses to visually presented letters and digits, both arbitrary visual shapes conveying culturally acquired meaning. Remarkably, this differential brain response to letters/digits occurred in a very fast presentation mode (< 170 ms per stimulus), low‐level confounds being controlled through random changes at every stimulus onset. These results suggest that already at the beginning of first grade, letters and digits give rise to specific neural responses, directly indicating selective modes of processing for one category compared to the other.

This novel finding goes beyond previous study where categorical responses emerged between familiar letters and unfamiliar pseudofonts (Lochy et al., 2016). At this age, children have had limited reading and arithmetic instruction, but they already possess letter and number knowledge due to early sociocultural and preschool‐related exposure to print (Wright et al., 1991; for digits see also Mejias & Schiltz, 2013; Hoffmann, Hornung, Martin, & Schiltz, 2013). Our results contrast with previous studies on the neuronal substrate of (differential) letter and digit processing in children. An fMRI study with 4‐year‐old preschoolers revealed greater selectivity for symbols (letters and digits) than objects (shoes and faces) processing in the left hemisphere, but no difference for letters versus digits (Cantlon et al., 2011). In a working memory task involving letters, digits or faces, 8‐year‐old children also did not show specific activation for letters versus digits (Libertus et al., 2009). Recently, an ERP study (Park et al., 2017) did not reveal lateralized response pattern for letters versus digits in 7‐ and 10‐year‐old children. Here on the contrary, inspection of topographies indicated that letters (vs. digits) are selectively associated with left occipital responses (PO9) and digits (vs. letters) to right occipital responses (PO10). In line with neuropsychological data (Starrfelt & Behrmann, 2011), topographies and amplitude values also suggested stronger discrimination abilities for digits than letters in 6‐year‐olds. While requiring further investigation, this might be because digits contain only 10 exemplars, and all (except 0) were presented here. In contrast, letters contain 26 exemplars, and only a subset was used (among which “J” and “Z,” not very frequent in French). Finally, digits might bear a meaning by themselves, already known at this age, whereas letters do not mean anything in isolation.

Digits in letter‐streams triggered right‐lateralized responses, whereas letters in digit‐streams showed a trend for a left‐lateralized response over posterior electrodes. The different lateralization patterns for the two visual categories suggest that specialization for these two culturally acquired symbols occurs preferentially in different hemispheres. Letters tend to be left‐lateralized, in line with the Phonological Mapping Hypothesis (Maurer & McCandliss, 2007), that posits rapid connections between posterior visual and anterior phonological regions. Digits are right‐lateralized, suggesting connections to right‐hemisphere structures, such as the parietal regions involved in nonsymbolic numerical magnitude manipulations (Cantlon et al., 2006; Hyde, Boas, Blair, & Carey, 2010), and from 6 years onward, in symbolic magnitude representations (Vogel et al., 2015). This finding supports the view that, at the beginning of learning new characters, specialization in the VOTC could be influenced by the nature of coactivated relevant representations and neural network during their visual processing (i.e., language and phonology for letters vs. numerical processing for Arabic digits). Indeed, distinct functional connectivity patterns exist between the VWFA and left temporal/inferior frontal cortices processing language on the one hand, and the visual number form area and the rIPS in the parietal cortex, processing numerical quantities on the other hand (Abboud, Maidenbaum, Dehaene, & Amedi, 2015).

Digits yield significantly stronger responses in the right hemisphere, whereas the amplitude difference between left and right hemispheres did not reach significance for letters. However, in other studies with the same paradigm, strings of letters led to significant left‐lateralized responses for third‐year kindergartners (Lochy et al., 2016) and first graders (van de Walle de Ghelcke, Rossion, Schiltz, & Lochy, submitted). Several reasons might account for this discrepancy. First, here we used single characters instead of strings, and the latter may increase the response level in parieto‐occipital regions (James, James, Jobard, Wong, & Gauthier, 2005; Park et al., 2014). Second, letters were presented in upper‐case instead of lower‐case, which might be more familiar, possess diacritic signs (accents, dots) and specific visual features (ascenders/descenders) enhancing responses. Finally, the contrast between categories was finer here than when letters were presented among unfamiliar pseudofonts.

Our results are congruent with fMRI data on adults (Park et al., 2011), where letters activated preferentially the left VOTC and digits a right‐lateralized occipital region. The discrepancy with developmental data collected in ERPs, which did not reveal specific lateralization pattern for letters versus digits until the age of 15 years old (Park et al., 2017), most probably originates in the sensitivity of the paradigm. Here, the fast and uninterrupted presentation of stimuli puts pressure on the visual system: each stimulus is visible for approximately 140 ms and is forward—and backward—masked by the previous and subsequent stimuli. No explicit task is required on the stimuli, avoiding the potential impact of task demands (Peters et al., 2015) and therefore the paradigm efficiently reveals automatic visual *discrimination* processes. Let us note that our paradigm does not quantify the potential common representation between the two categories but highlights the difference between them. Furthermore, it also reveals *generalization* over low‐level visual features in order for the response to emerge for one category over the other one. The greater sensitivity of our paradigm allows us to confirm that digits and letters are discriminated from each other and lead to specialized neural responses in 6‐year‐old children.

### Limitations and Conclusions

Our study has several limitations. First, in order to understand *when* specialized networks for these categories of symbols start to appear, it will be necessary to test younger children. Second, a larger sample and an extended assessment of literacy and numeracy skills would be necessary to assess if the amplitude of selective responses to letters and digits relate to character knowledge. Finally, although we demonstrate the possibility to use very short recording times (2 × 40 s), it would be beneficial to have more trials to increase SNR by averaging and therefore decreasing noise. Future studies should also attempt to quantify the frequency of occurrence of letters and digits in kindergarten children.

In conclusion, our study sheds new light on the visual cortex specialization for culturally derived characters like letters and digits and shows that already at 6 years of age, those arbitrary shapes are discriminated from each other. These findings agree with the proposal that VOTC specialization for cultural visual symbols partly depends on the relevant coactivated brain networks.
